# Health Planning in 1960s Africa: International Health Organisations and the Post-Colonial State

**DOI:** 10.1017/mdh.2018.41

**Published:** 2018-10

**Authors:** John Manton, Martin Gorsky

**Affiliations:** Centre for History in Public Health, Faculty of Public Health and Policy, London School of Hygiene and Tropical Medicine, 15–17 Tavistock Place, London WC1H 9SH, UK

**Keywords:** World Health Organization, Planning, Development, Africa, Health systems

## Abstract

This article explores the programme of national health planning carried out in the 1960s in West and Central Africa by the World Health Organization (WHO), in collaboration with the United States Agency for International Development (USAID). Health plans were intended as integral aspects of economic development planning in five newly independent countries: Gabon, Liberia, Mali, Niger and Sierra Leone. We begin by showing that this episode is treated only superficially in the existing WHO historiography, then introduce some relevant critical literature on the history of development planning. Next we outline the context for health planning, noting: the opportunities which independence from colonial control offered to international development agencies; the WHO’s limited capacity in Africa; and its preliminary efforts to avoid imposing Western values or partisan views of health system organisation. Our analysis of the plans themselves suggests they lacked the necessary administrative and statistical capacity properly to gauge local needs, while the absence of significant financial resources meant that they proposed little more than augmentation of existing structures. By the late 1960s optimism gave way to disappointment as it became apparent that implementation had been minimal. We describe the ensuing conflict within WHO over programme evaluation and ongoing expenditure, which exposed differences of opinion between African and American officials over approaches to international health aid. We conclude with a discussion of how the plans set in train longer processes of development planning, and, perhaps less desirably, gave bureaucratic shape to the post-colonial state.

This article explores the conception, elaboration and eventual fate of a series of health plans for five newly-independent countries in West and Central Africa in the 1960s: Gabon, Liberia, Mali, Niger and Sierra Leone. These plans constituted the major outcome of a World Health Organization (WHO) led technical programme, conceived with and funded by the United States Agency for International Development (USAID) during what US President John F. Kennedy had designated the ‘Decade of Development’. The health plans were intended to complement the economic development planning which post-colonial states were then avidly pursuing. They surveyed existing services and population needs, then made recommendations for extending institutional capacity and personnel, with the intention of systematically linking the expansion of social provision to the anticipated fruits of economic modernisation. Yet, it soon became clear that the plans had failed – their recommendations unmet and their priorities neglected by governments.

Why should this episode in international health politics interest us? After all, the countries under discussion were mostly small and rarely central to African historiography. It also may seem odd to pursue a case study in failure. There are two reasons. Firstly, we argue that the WHO/USAID West and Central Africa health plans (hereafter the ‘Africa plans’) were an important chapter in the emergence of what has come to be called ‘health systems strengthening’. Crudely this denotes the concern of international organisations with building health services, medical workforce and administrative structures in their poorer member states.^1^ Existing histories tend to locate its beginnings in 1978 and the Alma Ata Declaration, when the WHO announced a new agenda of universal access to primary health care, or in the early 1980s, when the World Bank began direct lending for health services.^2^ Yet, this is to overlook an earlier intellectual and policy history, which we propose to illuminate. Secondly, our study contributes to the larger history of development in post-colonial Africa. The narrative arc of this history is well-established, with the newly-independent states initially optimistic that economic growth could be accelerated through their active intervention. Yet, disillusion with development planning soon followed, with at best modest achievement, and at worst disastrous failure.^3^ Our example here adds the case of health and social policy to a literature hitherto concerned overwhelmingly with agricultural and industrial planning.

Our aim is to investigate what these planning exercises signified in the context of national independence and of global politics in the 1960s, what sort of development state the plans envisaged and what role international health organisations (IHOs) were to play in this state, and what forms of development the plans either foreclosed or made possible. We trace the circulation of these plans and the extent to which they contributed to and epitomised a particular form of developmental state in Africa, whose heyday was prior to the 1973 oil crisis. Turning to the texts themselves, we analyse elements of genre in the layout of the plans, as well as the demographic and economic fictions propagated in their production and circulation, assessing them as rhetorical exercises in relation to prevalent logics of development and modernisation. Finally, aware that the plans had little impact on the development of medical services, planning capacity or the evolution of health systems in any of the five countries, we highlight the specific forms of engagement that were envisaged and that emerged between IHOs and the post-independence state in Africa in the first post-colonial decades. We suggest that perhaps the most resilient outcome of this exercise was the commitment to planning processes themselves, whatever the outcome of their specific iterations. To begin though, we review the relevant historical literature on planning for health, at the WHO and beyond.

## 1 Historiography: Planning, Development and the WHO

What is known already from histories of the WHO about these first planning initiatives? Broadly, scholars have presented WHO’s early decades as the ‘era of eradication’, when ‘vertical’ (ie. externally managed) programmes addressing single diseases, like malaria, smallpox and tuberculosis, were the main focus.^4^ ‘Horizontal’ initiatives to build in-country health services only loomed larger following the Alma Ata Declaration of 1978, which set the agenda of universal access to primary health care.^5^ The early marginalisation of social medicine proponents, who favoured making access to services a core remit of the WHO, has been documented and explained partly by concerns of the United States about ‘socialised medicine’, partly by the promise of biomedical solutions like penicillin, smallpox vaccine and DDT.^6^ Also important was the incipient Cold War, born of geopolitical anxieties infusing American political culture in the late 1940s.^7^ Action against infectious diseases would produce, and then win hearts and minds to, a Western model of development as well as fostering the growth that would knit the ‘third world’ into global markets.^8^

Services, coverage and planning therefore featured initially as minor operational fields in early WHO literature, which instead emphasised the major disease programmes. The official history’s first volume (1948–57) detailed only the work of Geneva expert committees and field consultants, observing that no ‘rigid plan for providing medical care’ was advocated.^9^ Preliminary retrospectives similarly interpreted the WHO’s health services function as advising on administrative frameworks for public health.^10^ Volume II of WHO’s chronicle (1958–67) described the arrival of health planning as an aspect of economic development, with the Pan American Health Organization (PAHO) and various Latin American states as first movers, and the Fifteenth World Health Assembly (1962) the pivotal moment when planning for provision of ‘basic health services’ was endorsed.^11^ The five Africa plans were mentioned, though, without critical appraisal or contextual discussion of decolonisation, and they left no mark on the next official volume (1968–77), which lamented the region’s ‘limited experience in national health planning’ and the disconnection between economic planning and health.^12^ By now, the dominant narrative concentrated on the factors leading to the Alma Ata resolution, with the 1960s relegated in institutional memory to a precursor era, in which the WHO ‘lagged behind other sectors’ in rational planning methods.^13^ Finally, the Africa plans also went unnoticed in histories of USAID, where nation-building served as territorial containment of communist insurgency; instead, these traced the demise of Kennedy-era optimism to economic failures in India and conflict in Vietnam and Biafra.^14^

In the absence of discussion within the WHO historiography, then, how should we situate these initiatives? On the one hand, international health planning seems to have drawn partly on the contemporary policy vogue in the industrialised nations.^15^ By the 1960s faith in economic and social planning was ‘the political religion of post-war Europe’, stretching from the communist East to the social democratic West.^16^ At one extreme, ‘planning’ simply implied any state action with rational intent that intervened to modify laissez-faire capitalism: not much more than social and economic policy-making.^17^ At the other extreme was the ‘Five-Year Plan’ approach of the Soviet command economy, devised under Stalin in 1927–8 and adopted by communist China and Eastern Europe after 1945.^18^ This involved the setting of quinquennial investment and output targets (particularly for primary industrial products), and wholesale coercion of markets to achieve them. The democratic planning variant that emerged in the interwar West lay between these poles. For example, in Britain ‘planning’ was a feature of domestic policy debate, whose advocates ranged from the Conservative Harold Macmillan, to Labour intellectuals, to the ‘middle opinion’ think-tank Political and Economic Planning (PEP), whose 1937 report on the British health services famously called for a fully integrated system.^19^ Even the United States had modified its commitment to economic liberalism, for example through New Deal development plans, notably the Tennessee Valley Authority, and the success of the Marshall Plan in nurturing Europe’s recovery from war.^20^

However, given the African context, the WHO’s initiatives also need to be read as an aspect of post-colonial development planning. The historical literature on development, and planning within it, is voluminous. There is a broad consensus that this originated in the later colonial period, when empire’s ‘civilising mission’ gave way to a more active doctrine of trusteeship.^21^ French colonial theorists in the 1920s began to articulate the desirability of ‘un plan méthodiquement utilisé’ to achieve ‘la mise en valeur’ (enhancement/uplift) in their territories, while British contemporaries brought into the mainstream the idea of schematic ‘development’ (a trope originating with Marx, for whom it signified capitalist industrialisation).^22^ In practice this meant government loans or direct expenditure to improve productivity in the colonies and expand their market participation, not least because this would benefit metropolitan exports and employment.^23^ Scholars advance multiple reasons for the subsequent escalation of development activity: it was mooted as a salve to European depression; it reacted to both domestic and external criticism of imperial exploitation; it responded to African labour unrest and rising nationalism; and it gained momentum from a growing cadre of colonial bureaucrats and experts.^24^ Britain’s Colonial Development and Welfare Act (CDWA) of 1940 duly boosted funding and strengthened commitment to social objectives, as did France’s *Fonds d’Investissement pour le Developpement Economique et Sociale* (1946). It should be stressed, though, that colonial social expenditure levels were trivial throughout; Britain’s average annual colonial public health spend, 1929–40, was only £132 758, and even under the wartime CDWA, total imperial spending on medicine, public health and sanitation averaged only £646 790 per annum (1940–6), with West Africa probably receiving about one-third; these sums may be compared with London County Council’s public health budget in the depression year of 1931–2, of £8039 098.^25^

Historians divide on the extent to which these late-colonial foundations were the basis of the fully-fledged socio-economic planning of the post-colonial era. Some, with a view particularly to the Americas, trace a new paradigm to 1949, when US President Harry Truman’s inaugural address promised technical assistance for ‘underdeveloped areas’. They argue that as rhetoric this was distinct from both the ‘old imperialism’ and communism, and that as practice it furnished a humanitarian cloak for Cold War intervention, albeit in the guise of ‘a constant program for the better use of the world’s human and natural resources’.^26^ A cognate aim has been to expose the grip which ‘modernisation’ theory exerted on American social science and policy in the post-war period.^27^ Its classic text was Rostow’s ‘anti-communist manifesto’, which proposed a generic sequence leading to take-off into sustained economic growth that could be catalysed in poor countries by purposive action.^28^ Political science, sociology and psychology also proffered distinctions between ‘traditional’ and ‘modern’ societies to inform planners, before manifest failures and ideological critique exposed their hubris.^29^

Less US-centric scholars emphasise continuity across the end of empire and the diversity of intellectual and political currents informing development thinking. Thus, formal planning in India was initiated both by colonial administrators and by Congress politicians anticipating economic reconstruction after independence; its first Five-Year Plan (1950–5) bore similarities to such exercises under the Raj.^30^ In British Africa, post-war planning similarly sprang from the ten-year plans required by the Colonial Office from 1945 to ensure rational prioritisation of CDWA expenditures.^31^ Thus, Nigeria’s first Ten-Year Plan (1946–56) responded to these imperatives, as did its second (1955–60), now incorporating World Bank advice; its post-independence National Development Plan (1962–8) built on these.^32^ Nor can the tenets of development theory be characterised as a cascade from the West to colonial and post-colonial polities. Key economic thinkers were the West Indian W. Arthur Lewis, whose works on planning and on surplus labour as an aspect of industrial growth were touchstones, and the Argentinian Raúl Prebisch, who pioneered ideas of structural under-development in the trade relationship between ‘core’ and ‘periphery’ and the strategy of import substitution industrialisation to address this.^33^ Similarly, it had been independent Latin American nations that stimulated the debate about predatory private lending, which led to the establishment of the International Bank of Reconstruction and Development (IBRD) following the Bretton Woods meeting of 1944.^34^ Moreover, technical assistance for development was already underway at the United Nations (UN) before Truman’s call, led by semi-autonomous internationalist professionals in dialogue with Fabian, New Deal and European social democratic traditions.^35^

An equally diverse literature deals with the loss of faith in development planning amid perceptions of its failings from the 1970s. On the one hand is the critique of Western economic liberals, which invokes the chequered history of development aid to make the case for open markets, or at least for smarter aid strategies.^36^ Conversely, analysts on the left find vindication of the ‘under-development’ thesis, attributing disappointing growth to ongoing structural iniquities, and to the colonial legacies of artificially imposed states.^37^ Different again are anthropological accounts stimulated by Foucault’s insights. Such ‘post-development’ works go beyond the analysis of programme failures, to examine the discursive effects of the planning texts generated by internationalists.^38^ These asserted a universalising form of knowledge, which at once crowded out more nuanced understanding in the West and imposed itself on the subjectivities of recipients. Most crucially it was ‘productive’, regardless of its economic effects, as a form of bureaucratisation that foregrounded technical solutions to political problems, sweeping aside local knowledge.^39^ Other scholars of post-colonial *etatization* argue that this was abetted by nationalist politicians seeking both populist agendas after independence and income flows to sustain clientelism.^40^ The outcome was a ‘developmental state’, whose measures, analyses and bureaucratic processes responded to planners’ expectations, not least in the constant generation of (often dubious) indicators which demonstrated their suitability for financing.^41^

Finally, the handling of medicine and health within development historiography replicates the diversity of the broader literature. A longstanding Marxist tradition reads biomedicine as tool of empire, used by colonial rulers to manage labour in extractive industries and agricultural production.^42^ The post-colonial effects were to enshrine patterns of class inequality, with a Westernised medical elite prioritising curative services for favoured groups, to the neglect of prevention and rural health care.^43^ More recent scholarship decries the universalising tendencies of Western biomedicine, with its concealed assumptions about race and its arrogant dismissal of local knowledge.^44^ Despite this, health planning has a rather anomalous and under-explored status.^45^ Epidemiological evidence linking life expectancy gains to development is fairly robust (the ‘Preston curve’) and probably attributable to the transfer of biotechnologies like vaccination, antibiotics and oral rehydration.^46^ And while it is reasonable to point to some achievements of local healing practices – the derivation of variolation from folk medicine, for example – post-development thinkers do not suppose that mass societies may control communicable diseases without biomedical interventions.^47^ Moreover, ethnographic investigation denies easy distinctions between traditional and allopathic medicine in settings where both flourish; instead understandings prove fluid and practices flexible.^48^ All this cautions against uncritically situating health planning within narratives of the rise and fall of development.

To summarise, histories of the WHO have acknowledged the existence of planning for health services in 1960s Africa, but have not subjected it to critical scrutiny. How it emerged, and how international experts positioned their endeavours in the context of the post-colonial, Cold War moment remains to be resolved. The question of why it failed is also obviously germane, given the negative retrospectives on planning, although we are limited in what follows to circumstantial evidence from secondary sources. The literature also invites us to consider the ‘productive’ effects of health planning discourses. Even as it failed in its nominal goals, what was its shaping effect on the developmental state? It is the interrogation of this relation, between state capacity, international governance and the health needs of ordinary Africans, which now concerns us.

## 2 Groundwork and Contexts for Planning

Somewhat in advance of President Kennedy’s designation of the 1960s as the ‘Decade of Development’, the UN had envisioned a transition ‘from protection to development’ in international policy.^49^ The original basis of intergovernmental working had been as guarantor of security, workers’ rights and freedom from epidemic disease, but this had given way to that of development, in which the different UN agencies would work together for the ‘basic economic and social needs’ of poorer countries.^50^ This built on a move towards planning for development, which, as we have noted, represented not simply a late-colonial technology of control, but also an established mode of domestic governance in industrialised nations.

In this arena, and with a focus on what Gunnar Myrdal termed ‘underdeveloped regions’, the WHO’s anticipated role would fall into three areas: strengthening national health services; eradication of malaria and smallpox; and training of medical and auxiliary personnel.^51^ For the former, the emphasis was on progression from specific disease programmes towards planned co-ordination of preventive and curative services; these were to achieve ‘maximum availability’ for the ‘entire population’ ‘of a well-balanced and integrated national health service’.^52^ Collaboration with the ‘United Nations family’ to align health with national economic planning was welcomed.^53^

What form could such health planning take? By the early 1960s, colonial articulations of development needs and priorities had already come to lack legitimacy and traction, both within newly-independent countries, and on the international stage constituted by the UN and its agencies. The UN increasingly reflected the skew towards new members, as well as the exigencies of Cold War polarities. For the UN system, the early 1960s was a high point in its moral authority, at a time where the record and conduct of colonial stewardship was routinely maligned, and the clampdown on Civil Rights protest undermined the status of the USA among African nationalists.^54^ UN agencies projected themselves into the rhetorical space of post-1945 international politics as organisations tasked with implementing the collective wishes of member states, in a series of agreed technical interventions and as expressions of consensus where such might emerge. The WHO made use of this rhetorical space to present its shaping of an international agenda around health planning as a response to purported historical imperatives.^55^

There was, of course, a strong internal political dynamic at the heart of this agenda, which was gradually consolidated through the 1950s under the aegis of the WHO Expert Committee on Public Health Administration, and bore many of the hallmarks of 1930s social medicine through the impact of committee members such as James Mackintosh, founder of the Society of Social Medicine, and Andrija Stampar, a veteran both of the League of Nations Health Organisation and of the development of health centres in Yugoslavia.^56^ The committee sought to discern the functional components of health administration and authority, and identify the potential for c-oordination of health care delivery at a national level. It was presumed that co-ordination would be beneficial, in terms of both cost and efficiency, and, by the early 1950s, the WHO sought to position itself as the body responsible for filtering expertise on co-ordination into advice for national health planners.^57^

The WHO’s initial forays into planning assistance were at once pragmatic and top-down; surveys of local services and needs provided the data on organisation and personnel which could then be folded into a prescribed plan. The pattern of privileging the skills and knowledge of the outside expert, noted by Randall Packard in the context of the Global Malaria Eradication Programme, was set early in the history of the WHO, in spite of a stated aspiration that local conditions be factored into all assistance programmes.^58^ The committee sought to elaborate both the principles and preconditions of health planning, which would be predicated on availability and standardisation of relevant data, be a declared object of national policy, and be comprehensive across the territory, population and breadth of service provision. The clearest concerted application of emerging planning rationales in the context of international development came from Latin America, where the PAHO pioneered a planning methodology named after the Centre for Development Studies (CENDES) at the Central University of Venezuela.

The PAHO-CENDES model – applied across Latin America in the 1960s – linked together development priorities, the reengineering of state planning capacity, statistical analysis and a flexible approach to framing and meeting health priorities. Its holistic approach to planning as development was appealing to national governments and international organisations, banks and donors alike, but the demands of the methodology proved too onerous for most states; its most significant contribution was in its signalling of the potential for aligning development and planning as part of state apparatus in Latin America and elsewhere, as evidenced in a succession of international conferences on health planning in the developing world held not only in Latin America, but also in Manila in 1964 and Addis Ababa in 1965.^59^

## 3 Health Planning for Africa Begins

How would this assemblage of ideas and approaches apply to Africa? Throughout the first six decades of the twentieth century, colonial Africa had remained largely invisible to UN agencies and their League of Nations predecessors. The British CDWA of 1940 was passed with the explicit aim of forestalling international interference in Britain’s colonial territories, while statistical measures of science and economic performance in Africa had been established by the major imperial regimes.^60^ WHO intervention and capacity in Africa prior to national independence (beginning with Ghana in 1957, and rapidly unfolding across the continent in subsequent years, with seventeen countries becoming independent in 1960 alone) was extremely limited, with assistance to Yaws eradication programmes, piloted by WHO and financed by the United Nations Children’s Fund (UNICEF), perhaps the most significant early contribution by UN agencies to health services.^61^ In evidence of its opacity from the perspective of the WHO, Africa was not to be covered by the Global Malaria Eradication Programme, due to presumed technical difficulties and under-interrogated notions of acquired immunity.^62^

African countries were to benefit, however, from the supplementation of existing international assistance programmes with ‘national plans’, as approved at the World Health Assembly in 1962. For the African case, negotiations began with the United States government to provide the extra funding that would be needed.^63^ At first it was envisaged that ten African countries would be the first subjects of national health planning, and this was explicitly linked to their recent achievement of independence.^64^ The initial phase of the exercise, devised by Fred Grundy and Milton Siegel in 1962, was the recruitment of ‘senior public health men’ to act as consultants, ideally recently retired so that they could give a long-term commitment, and the development of a training programme to prepare them.^65^

It was clear from the outset that the exercise was intended as a pilot, not only of health planning in Africa, but of the WHO’s capacity to spend earmarked development funding. The significance of the process, as opposed to the long-term outcome, is captured in the tone of internal correspondence on the nascent process:

We believe that if we can do this job well and do it fast we may look forward with confidence to receiving yet greater financial support… We recognise the importance of this opportunity, and consider that WHO can make a success of this work. We have not as yet of course approached any African countries which are possible participants.^66^

The opportunity presented by new nations was to be matched by a rhetoric of vigour and enthusiasm, and met with attributes of skill, commitment and drive.

USAID approved funding of $500 000 and national visits were made in West and Central Africa to select the countries that would receive the intervention, alongside concurrent national economic planning.^67^ Some, like Upper Volta, bluntly responded that WHO’s offer ‘would not be welcomed’, while others, such as Cote d’Ivoire, had already approved a ten-year development plan designed by France, the departing colonial power.^68^ It was eventually agreed that five countries would be subject to the programme, Niger, Gabon, Liberia, Sierra Leone and Mali, with prospective extensions to East and East-Central Africa.^69^

It would be wrong to characterise WHO’s approach as simply a hubristic imposition of Western values and ideas onto post-colonial states. From the outset, the Executive Board was alert to the importance of letting national governments articulate their own needs, with the UN agencies advising on how to ‘co-ordinate and balance’ those needs, and furnishing expert advice to build administrative capacity.^70^ The training course for consultant planners was to include not only administrative skills, but also ‘social and anthropological’ analysis of local cultures and health behaviours, as well as comparative awareness of the varied models of national health systems, including pluralist, socialist or welfare capitalist arrangements.^71^ The course itself was to be delivered at the University of Michigan by Nathan Sinai, a pioneer health services researcher and veteran New Deal liberal.^72^ Internal discussion at regional level also registered country representatives’ concern to avoid ‘a kind of new intellectual colonialism’ imposed on recently liberated nations.^73^ This position did not simply reflect Cold War division, but a broad-based view that the planning consultant in the field should not ‘carry his nationality with him’ when advising on system design: ‘How is a Russian to advocate capitalistic medicine or an American socialistic medicine if that were necessary and appropriate.’^74^

Despite this reflexive caution, cultural and political frictions were evident from the outset. WHO staff were expected to work at a speedier pace than national officials, who duly felt themselves being ‘bulldozed’.^75^ Observation of early post-independence initiatives, such as extravagant but ill-considered building projects and bureaucratic graft, also suggested there would be obstacles to successful planning.^76^ Nor was financing discussed in a substantial way as integral to this activity. Although the UN officially encouraged inter-agency working, the WHO Executive Board made no effort to revive its earlier collaboration with the International Labour Organization (ILO) on medical aspects of social security in the health planning venture.^77^ This neglect was despite the growing expertise in this area on the part of ILO officials, such as Laura Bodmer, and WHO advisers, such as Brian Abel-Smith, and presumably reflected the ongoing political sensitivities surrounding health system financing.^78^ Instead, the creation of integrated national health services in Africa was understood in terms of establishing the requisite administrative structures, workforce and institutions to meet population health needs. The expectation was that donor support would begin the process, aided by limited national resources, which would gradually scale up as economic development took hold. Thus, the first five years of Sierra Leone’s national plan, including health, were costed at £100 million, of which it was assumed about a third would come from national budgets and the remainder from ‘any outside source’.^79^ Yet, the question of how national governments were to build health financing structures was never properly confronted.

## 4 The Plans: Genre and Statecraft

The mid-1960s was the high-tide of optimism about WHO’s health planning in Africa. By 1965 the expert consultants were in place and the five plans nearing fruition. Four phases had been mapped out: beginning with a ‘fact-finding stage’ leading to priority setting; followed by the planning stage itself with its consequent reorganisations; next would come an evaluation; and finally training and capacity-building so that the cycle would continue.^80^ WHO officials reported to USAID that health was now successfully embedded in the larger economic plans, and that the exercise had made a positive contribution to state-building, although administrative and statistical capacity still remained weak.^81^ The plans themselves were published more or less on schedule, with Gabon’s (covering 1965–80), Mali’s (1966–76), Niger’s (1965–74) and Sierra Leone’s (1965–75) all in 1965, and Liberia’s (1967–72) slightly later, in 1967, to coincide with its five-year economic development plan.^82^

Of the countries that accepted USAID funds and WHO technical assistance for health planning, some clearly identified an opportunity to fund an already existing planning programme.^83^ In Liberia, the prominent presence of an existing USAID Mission made it easier to secure government assent to the WHO/USAID plans, despite the WHO representative only managing to meet subordinate officials on his visit.^84^ While the Ivory Coast and Upper Volta (now Cote d’Ivoire and Burkina Faso, respectively) rebuffed WHO/USAID overtures, three francophone states – including Mali, with its socialist government – incorporated the WHO-led health planning programme into national planning regimens.

In each of the countries, national economic and budgetary planning was at a preliminary stage, even where late-colonial, early independence or ongoing development plans had already been elaborated. For Niger and Sierra Leone, the advent of national independence had greatly reduced capacity in health care, underlining the weak colonial commitment to training indigenous health workers. For Niger, planning was expressly conceived as an opportunity progressively to abandon colonial forms, and to align national development with the needs and possibilities of the new nation-state.^85^ Liberia and Gabon, with political continuity and resource wealth respectively, and Mali, a centre of French administration in Africa, fared somewhat less badly from an organisational perspective, but state planning nonetheless augured a new relation between the state and its population and resource base.

For Niger, the balance of payments was the key indicator and target for economic planning; given the weakness of the industrial sector and reliance on agriculture of much of the workforce, this balance continued to be negative. State bureaucratic capacity was also weak, with the health planning team mourning their reliance on national accounts which were already three years out of date in 1964.^86^ In Mali, planners navigated a narrow political space between the apparatus of state inherited from the French, and the various popular movements allied to the ruling party, of whom the youth and women’s movements were seen to be particularly preoccupied with health, welfare and education. The question of manpower was pressing in all countries. At the time of publication of its health plan, Liberia was undertaking a preliminary manpower survey with the assistance of the ILO, and the government was unable to identify the employment sectors of school leavers.^87^ Nonetheless, health planning teams were keen to stress the coherence of national planning, and the convergence of planning in health with economic programmes.^88^ While we have no specific evidence as to the reallocation of manpower resources required to produce health plans, or the fates of the bureaucrats involved, each plan suggests a close dialogue with economic planning teams. Via USAID funding and WHO expertise, the politics of public health entered the national sphere.

The plans had a clear political function. Their composition and organisation invoked and reproduced clear lines of authority in health, from the minister of (public) health, down. The fate of health governance was tied specifically to the economic needs and output of the country, not only through explicit commentary, but through rhetorical and organisational flourishes which echoed national plans. We see, after a brief preamble by the sitting minister of health, that the Sierra Leone plan proceeded for a third of its length through an enumeration of the general characteristics of, first, the topography, climate, industry and natural resources of the nation-state, next its population and gross economic indicators, and, finally, legislative and administrative capacities and access to educational opportunity. It then outlined, in turn, the ‘health situation’, health organisation and administration, physical and personnel resources in health, other health activities (namely, control of communicable diseases, environmental hygiene, housing, occupational health services and health education) and the health budget in relation to national expenditure. The final third of the document proper appraised the health problems of the country, determined the relevant priorities, defined plan objectives and, in two short chapters, outlined the ‘Plan of Action to Be Developed’ and the ‘Definition of Plan and Budget’. There followed detailed cost estimates for specific building works at existing hospitals, together with an addendum outlining new priority fields not already outlined in the plans: most of these comprised additional wards, technical apparatus and staff housing.

The plans for Niger and Mali proceeded in much the same way, with a preface by the minister with responsibility for public health, and an elaborate presentation on state capacity, which was stronger on administrative structure than on economic, demographic or health data.^89^ It focused on the status of health services, the most urgent health priorities, the available budget and a plan to address these given current and projected state and development aid resources. Without the data to describe or predict economic and population activity with precision, a variety of assumptions about population growth, internal and external migration and economic activity underpinned the exercise of revenue generation, national planning, and grandiose projections for investment in health. Pragmatic questions aside, the presentation of the plans themselves raises interesting issues of style, content and audience. In the structuring and ordering of the plans, we can discern a clear politics, deeply technocratic in its essence, as well as utility in projecting the authority and pre-eminence of the ministry in determining outcomes in health.

The Sierra Leone plan grappled with the mismatch between the ideal health service and the status of medical and health capacity in the country. As a former British colony, it had inherited a patchwork of missionary provision, colonial government health care, private clinics and unintegrated technical services in laboratory provision and disease surveillance, together with historic underinvestment in training of local health workers. Existing provision guided funding and planning decisions, such as where to locate the hub of a national laboratory service, even as planners dreamed of a new system at once consolidated in new campuses and distributed equitably across the national space.^90^ Demonstrable arbitration of feasibility was a constantly revisited refrain; plans highlighted the existence of ministry of works templates for building clinics and hospitals, and made much of the provision of simplified and portable plans and cost estimates, an emphasis on flexibility, deferrable costs, re-use and conversion, and careful attunement with existing plans and manpower estimates.^91^

It is beyond the scope of this investigation to interrogate the production of the statistical measures that underlie the planning documents, though the cautions of recent scholars about the faulty metrics of African economic and political performance are obviously salient.^92^ Indeed, this caution is foreshadowed in the preparation of the plans themselves, which often give an insight into the mechanics behind the production and circulation of partial and inaccurate figures. The Sierra Leone plan, for instance, notes that vital statistics suffer from non-compulsory registration of births and deaths. In this case, the UN Demographic Yearbook statistics reflected only Freetown – the capital – and environs, where the presence of a maternity hospital skewed the validity of already partial statistics.^93^

In summary, the plans were not able to be methodical, and strove instead for an ostensibly pragmatic assessment and projection of state capacity in health. Goodwill, close involvement of national civil servants and a cautious approach to budgeting were not sufficient to overcome deficits in regulatory, statistical, administrative and national planning apparatus, not to mention the absence of a guiding methodology for health planning.^94^ The result was that, for each plan, the proposed activities closely followed the lines laid out in a tabulation of existing resources. The logic of multiplication and reinforcement predominated throughout, with an emphasis on more hospitals, more expertise, better training. The resulting documents leaned heavily on overarching national plans, offered as the framing rationale and source of data in the opening sections of each published health plan.

In these instances, the importation of a donor-specified template for health systems elaboration and planning into bureaucratic and information systems (civil service and national statistical measures), ill-equipped to match planning logics to the capacity to implement policy desires, led to an involution of bureaucratic shortcomings into the heart of supposedly vigorous and hopeful commitments to newly-independent state planning under the guise of pragmatism. Although ostensibly successful, in that plans were published for each of the five pilot states, the degree to which the plans were intertextual with already insufficient late- and post-colonial state-bureaucratic productions,^95^ and pressed into the projection of statehood and independence in the arena of international public health, is significant in understanding how the fates of the plans become independent of their measurable health outcomes. The consequent intertextual amplification of poor data, impressionistic echoing of material from national plans and the series of platitudes and caveats operated more as a means of matching health bureaucracy to health services (thus extending at least the notional basis of governance), and thus operationalising the discursive effects of planning, than as an effective programme for identifying and meeting the health needs of the population.

## 5 Outcomes

In 1965, to build on this apparently successful process, WHO convened a conference in Addis Ababa, which aimed to derive some generic lessons from the work thus far. It discussed, for example, the issue of data requirements for planning, appropriate administrative structures and modes of liaison with finance ministries and, crucially, whether any general methodology was now emerging.^96^ Meanwhile, in Geneva successive expert committees were convened, the first in 1966 to report on the work thus far by both WHO and PAHO and propose some common approaches, and the second in 1969 to study and systematise training for planners.^97^

By the late 1960s, however, it was clear that something was wrong. A hiatus had ensued after the plans were published, with implementation assumed to be proceeding, and it was not until mid-1968 that progress and evaluation began to be discussed. The WHO Africa Regional Office (AFRO) provided the preliminary assessments, but its reviews were cursory and discouraging.^98^ They revealed that only two of the countries so far had made any attempt at implementation, with the principal difficulty claimed to be lack of qualified staff to take them forward.^99^ In Sierra Leone, recruitment and capital expenditure had ceased from 1965 ‘owing to a financial crisis’ and only three new health centres had been opened, with donor funding.^100^ In Mali, the aim of increasing doctor numbers from fifty-two to eighty-six and pharmacists from six to fourteen fell short by thirty doctors and seven pharmacists, and the planned new *Institut de Biologie* and *Ecole des Assistants Medicaux* had not been built, although there had been nine new health centres and nurse numbers had risen, particularly of *brevetés* (below diploma level).^101^ Liberia had produced ‘no operational report’.^102^ Niger had failed to deliver the five new hospitals, four hygiene departments and ten rural dispensaries proposed in the plan, and the forecast health investments for 1965–7 of 998 million CFA francs had in reality been 104 million.^103^ How, wondered WHO’s Assistant Director-General (ADG) John Karefa-Smart, could these governments:

… be persuaded better than we have succeeded in doing until now, not to put the nicely printed plans away in filing cupboards, but use them actively in the development of their health services[?]^104^

Why should the results have been so lamentable? We have not been able to examine directly the records of the five states, so our answers are speculative. However, the secondary literature offers ample evidence of the challenges which they faced. In socialist Mali, economic planning was premised on rapid productivity increases through cash-crop farming and new food-processing industries. These did not materialise, and in the meantime loose monetary policy and heavy public expenditure fuelled foreign indebtedness and inflation. Popular unrest and repression followed, stirring political crisis and a military coup in 1968 that overthrew President Modibo Keita.^105^ Gabon had similarly entered a phase of political turbulence as President Leon Mba sought to consolidate autocratic power in place of the multi-party state created at independence; a coup attempt in 1964 led to French military intervention. Planners’ efforts at rural regeneration from fruit exports had failed through neglect of local interests and poor communications, even as the proceeds of France’s investment in oil, manganese and uranium furnished the state with resources for clientelism.^106^ The other ex-French colony, Niger, was mostly landlocked desert subject to climate instability, with a small export market in groundnuts and one of the lowest growth rates in Africa: recorded GDP from the mid-1960s was either flat or falling.^107^ Nor was there democratic pressure for social spending. President Hamani Diouri established authoritarian rule, suppressing the Sawaba party that had sprung from Niger’s labour movement, and used the scant revenues to reward ethnic or regional loyalists.^108^

The other two nations were richer in resources, but also confronted political impediments to health planning. Liberia’s independence dated to 1847, but, thanks to bountiful rubber and iron ore resources, it was effectively a ‘gatekeeper’ state, mediating American industrial and strategic interests.^109^ Its president, William Tubman, was in office from 1944 until his death in 1971, exercising one-party rule with emergency powers to suppress internal dissent, as he did in 1966.^110^ Although buoyant rubber prices sustained growth through the 1960s, this was unevenly distributed, with wealth highly concentrated among the minority Americo-Liberians in the coastal region and Monrovia. Planned distribution of health resources was at odds with Tubman’s preference for patronage in rewarding the loyalty of local rulers in the interior.^111^ Similarly, the politics of independent Sierra Leone, where the nationalist parties depended on the support of traditional chiefs, was also structured around patron/client largesse dispensed on ethno-regional lines.^112^ Initially, growth had risen, driven by the diamond economy, but declined from the mid-1960s, as plans to develop commercial agriculture faltered.^113^ This prompted political turbulence, with serial military coups in 1967/8 ousting the prime minister, Albert Margai, in favour of the opposition leader, Siaka Stevens.^114^

It is somewhat ironic, then, that it was a Sierra Leonean, John Karefa-Smart, who had to deal with the fallout as WHO’s ADG (1965–70). Karefa-Smart had arrived in international health after studying medicine at McGill and public health at Harvard, having despaired of the ineffectiveness of local missionary medicine, which he saw as self-interested rather than preventive.^115^ After spells teaching public health at Ibadan Medical School, then working with WHO AFRO in Liberia, Nigeria and Congo (1951–4), he had entered Sierra Leonean politics just as the independence movement gained momentum.^116^ Under the first post-colonial government (1961) led by Sir Milton Margai, he became foreign minister, but quit the country in 1964, following Margai’s death and the succession of his more authoritarian brother.^117^ His political exile was prolonged following the coups, as the new prime minister, Siaka Stevens, was a long-term rival.^118^ Freed to undertake the WHO role, he had burnished his credentials championing African nations’ rights at the UN, extolling the development/assistance project and articulating a non-aligned position for Africa within Cold War politics (although personally believing that ‘community health and capitalism may well be antagonistic’).^119^ Thus, he was at once convinced of the planning agenda while also painfully aware of how this could be disrupted by political turbulence and economic failure.

By late 1968 pressure was mounting on Karefa-Smart and on Basile Adjou-Moumouni, AFRO’s Beninese deputy director, to deliver a resolution to the planning exercise.^120^ On the one hand USAID required the remaining money to be accounted for and spent, and on the other full evaluations would be needed following the lacklustre start.^121^ Anticipating criticism, Adjou-Moumouni initially lobbied to have these conducted by regional officials rather than outside consultants, who would presumably be less sympathetic.^122^ He also urged that in place of evaluation should be a milder ‘review’ or ‘appreciation of the way the plan was being put into effect’.^123^ Karefa-Smart meanwhile attempted to put the grant surplus to a use for which it was not originally intended: the organisation of seminars in health administration for all the African countries in the region – essentially resequencing the ‘stages’ so that the further training assistance preceded the evaluation and expanding beyond the five beneficiaries.^124^ Although neither of these prominent African health diplomats were involved in devising or implementing the health plans, each was keenly aware of the ways in which post-colonial planning was implicated in the growing pains of post-colonial African governance. The solution offered by the African parties, then, was to avert strict assessments which would cast novice governments in a poor light, to maintain recipient autonomy over donor funds and to devote those resources to building internal administrative capacity.

**Figure 1:**
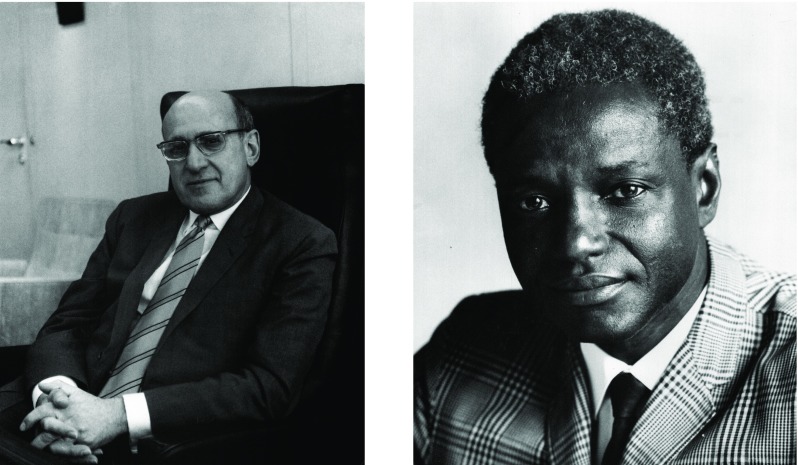
Milton Siegel (left); John Karefa-Smart (right), leading officials of the WHO active in the debate on the evaluation of the Africa health plans. (Left: WHO Archives photo ref. WHO_14661 – Caption: Milton P. Siegel, Assistant Director-General of WHO. © World Health Organization/Tibor Farkas. Right: WHO Archives photo ref. WHO_11980 – Caption: Dr John Karefa-Smart, Assistant Director-General of WHO. © World Health Organization/Tibor Farkas.)

This more relaxed approach was quickly scotched by Karefa-Smart’s fellow ADG, Milton Siegel, an American Mid-Westerner whose WHO track record had been in the Division of Administration and Finance, and who had been partly responsible for devising the consultancy component of the Africa National Health Planning programme (Figure 1). Diversion of funds breached the original USAID agreement, so could not be countenanced, and evaluation had to be accomplished by experienced consultants, though it was accepted that these might be from developing countries.^125^ After a protracted search for appropriate individuals, the evaluations finally began in 1970 and the resulting texts painted more copious pictures of plans unfulfilled.

Only the Liberia evaluation survives in the WHO archive, which is unfortunate since this country was almost certainly the most successful of the planning pilots. It received substantial amounts of American aid in this period, thanks to historic ties dating to its founding for repatriated Afro-American slaves and to its anti-communist stance. As noted, it was politically stable under the rule of President Tubman, and enjoyed some growth from primary exports. Despite these advantages, and although a mechanism for health governance was slowly coming into place, the consultants clearly felt it had fallen short:^126^ health had suffered a declining share in national expenditure; necessary appointments of local public health directors had not been made; there were insufficient technical personnel like midwives and community nurses; and, consequently, the anticipated disease control and eradication programmes were languishing.^127^ Capital expenditure had been lavished on the J.F. Kennedy Memorial Hospital in Monrovia, without consideration of the recurrent financial commitments expansion would necessitate, while medical training was centred on a Vatican-sponsored school that gave insufficient priority to preparing Liberians themselves to join the teaching faculty.^128^ Meanwhile, booming Monrovia had no municipal public health authority to address vital issues of sanitation and preventive medicine.^129^ Finally, no capacity existed for epidemiological or health statistics gathering, leaving politicians with ‘no basis for reasoned decision-making’.^130^

A final verdict on the African national health plans was delivered by WHO officials in the early 1970s. Despite the ‘goodwill shown’, and the buy-in of national staff, it was conceded that ‘evaluation disclosed that the implementation of the plans recommendation [*sic*] showed little progress’.^131^ Not only did this demonstrate that ‘the plans were too ambitious’, it also revealed the ‘prerequisites for health planning’, in the form of regulation and administrative machinery, were mostly ‘not present’. Moreover, there were as yet no established ‘scientific methods’ of planning, hence the approach had been ‘pragmatic’ (presumably a tacit admission that without proper epidemiological data the labour and staffing projections were mere guesswork).^132^ This diagnosis echoed the comparative judgement of WHO officials, contrasting health planning in Africa with more systematic methodologies emerging elsewhere:

… the ‘pragmatic’ approach… based on the experience and intuitive skill of the planner… [rests] heavily on judgements not reinforced by data and systematic analysis, [and] is relatively vulnerable to political pressures that are not oriented to the long-term welfare of the whole country.^133^

Alongside the practical difficulties arising in the evaluation process, and the barely concealed antagonisms that they represented, were others that arose from the economic and cultural distance between the planners and their subjects. In the absence of financial resources, it was asked how could the African nations realistically develop the necessary expertise? And were not concepts like priority setting, ‘planning, programming, plans of action… and integration’ all Western bureaucratic modes, which were said to be poorly understood by ‘responsible and executive authorities’?^134^ Ironically, practices at WHO and USAID were little better. By 1973 the remaining budget of $130 000 had still not been spent, despite the ‘painful’ argument five years earlier between Siegel and Karefa-Smart over training for capacity-building, and no one in either organisation could now recall the original terms of agreement, to the despair of more recently appointed staff.^135^

## 6 Discussion

The events we have observed here emphasise the tactical and improvisational nature of the planning exercise for African civil servants; indeed, these tactics brought about a planning regimen more concerned with a series of intellectual and rhetorical feints than with fidelity to the match between resources, capacity, needs and development. As Mabogunje has tellingly argued (with reference to urban planning, but with broad applicability), the planning process in post-colonial Africa resembled ‘[trying] to *run* with the majority of its population… while not indifferent to *hunting with the hounds* of domestic and international capital’.^136^ One of the key purposes of planning was to project a capacity for self-government into an international arena, producing at the same time a civil service cohort and a larger planning process. Available numbers, simple stories and portable typologies were pressed into service time and again in rendering states open to processes of international governance. This arena was constituted in part by the WHO, one of the most amenable and well-supported avenues for the assertion of the legitimacy of post-colonial states.^137^ Indeed, mooted WHO training for health planners in the early 1960s, although not limited in scope to Africa, was explicitly linked to the newly-achieved independence of African states.^138^

Even at the time, insertion of health plans into broader programmes for the generation of economic and national plans raised anxieties about the purpose and fates of such enterprises. The colonial ‘plans’ which acted as precursors to the planning exercises of the 1960s bore little resemblance to processes set in train in Europe and North America in response to economic crisis and war in the 1930s and 1940s. While France and Britain pursued ostensibly distinct regimes of planning and development in the immediate post-1945 period, planning had little coherence, and even less statistical underpinning. Indeed, IBRD economic surveys of the late 1950s, in territories under British colonial rule, formed the backbone of planning intelligence in some post-colonial states, and served simply and directly as a conduit of Colonial Office data into post-colonial settings.^139^ Very rarely was post-colonial economic intelligence free of the taint of instrumental data-gathering on the part of imperial regimes.

Within the largely unsystematic modernising discourse exemplified in the post-colonial developmental planning process, health and medicine were key to translating the political ambitions of early independence leaders into popular legitimacy. Health and medicine enabled a new post-colonial civil service to translate national aspirations into technocratic process. As a contemporary WHO-commissioned report noted:

There has been a genuine desire to have something to show for independence and therefore a temptation for African political leaders to allocate resources to social services and facilities on a scale that commits their countries to living beyond their means, Accordingly, the health planner is likely to receive more encouragement from political leaders than from his colleagues in charge of planning economic development.^140^

As Ben-Amor and Clairmonte, two leading participants in the co-ordination of planning across post-colonial Africa in the 1960s, note: ‘some plans… are nothing more than a collection of official statements embodied in a document, which once published is destined for the archives, or deferentially referred to occasionally in a ministerial declaration.’^141^ These authors also draw attention to the vanity, self-referentiality and divorce from practical decision-making and evaluation of many of these exercises. Caught between piety and urgency, the plans themselves assumed a largely fictive role as rhetorical instruments that figured and brokered the capacity of a poorly-equipped state apparatus in its attempts to grapple with the inheritance of an unfit colonial apparatus. In this sense, the minor sectoral planning enterprises in five small West and Central African nations examined here are exemplary of the fates of the planning enterprise, if not exhaustive with respect to sectoral and service management in post-colonial health in Africa. ‘Planning in Africa,’ the same authors note, ‘is intimately wedded to political freedom and has become a weapon in the war against the forces of under-development.’^142^

This story sits alongside another narrative of African contributions to international health governance that has many of the same roots in post-colonial health administration and planning. In this story, figures such as Thomas Adeoye Lambo, John Karefa-Smart and others, African doctors who became prominent in international health politics in the 1960s and 1970s, demonstrate how the health needs of African states and communities become central to questions of equity, health governance and international health diplomacy in the Cold War period.^143^ At the same time, Africa continued to be portrayed as a domain within international health to be acted upon, rather than generating its own consolidated and recognised contribution to ideologies of planning.^144^ As we have seen, the professional and diplomatic trajectory of John Karefa-Smart exemplifies an indirect feedback from the WHO/USAID health planning exercise in 1960s Africa, illustrating some of the thornier issues of stewardship, responsibility for development and political authority in international health emerging from the early post-colonial experience of health planning and its shortcomings.

## 7 Conclusion

This coda, and the rather dismal assessment which preceded it, would seem to condemn the WHO’s Africa health planning venture to a historical verdict of failure. Indeed, it might even serve as a textbook case for sceptics of the development project, who castigate the ‘central planning mentality’ as ultimately ineffective because it rests on utopian foundations.^145^ Such a reading would, though, be reductionist, not only because no definitive means of judging long-term impact exists outside of the actors’ own impressions, but also because it neglects the broader themes which it can illuminate. Certainly, the forgotten fates of these plans emphasise a proliferation of projects across a poorly articulated, post-colonial planning space.

The plans described in this article were unsatisfactory, as attested by local and international evaluators, civil servants and funders. All the same, WHO internal evaluations noted the stimulus effect of training and of a disposition towards planning, as well as increased consciousness of ‘good administration and proper management of health services’.^146^ There was a clear effort to make these plans circulate, commenting on their potential shelving as an outcome to be forestalled, and convening meetings and conferences to give the planning exercise some much-needed traction.

Through the 1960s, the means to analyse the inputs and outcomes of the planning exercises were clearly insufficient to the task; however, what we do know about the framing, production and reception of the planning process and its paper outcomes epitomises much of what has come to be understood of African development. The reification and recuperation of results and processes, however scant and unsatisfactory, is a recurrent feature of development logics in post-colonial Africa. Results and processes generate meaning in development; they have effects in governance, in donor relations and in policy-making, not to mention in staffing and personnel within country civil service and international organisations. These processes become instantiated in policy and personnel, conferred with the patina of expertise, while the numbers take on a life of their own, untethered from and heedless of their problematic and unsatisfactory origins.

The African post-colonial nation, in its guise as developmental state, was the space *par excellence* in which planning could come to occur. In the same nations, inequality and incapacity emerge in sharp relief, the ironies of the settlement in which certain territories become silos of impoverishment, are progressively laid bare and then repeatedly attached to the cultural deficiencies of the states in question. James Ferguson’s reminder, that cultural difference only becomes analytically salient when it exposes deep inequity, prompts our attention to the processes by which narratives of African failure took root in the period of de-colonisation.^147^ The fate of the USAID-funded WHO health plans of the mid-1960s is a clear illustration of these processes.

And yet, these ‘failures’ were productive. As Ferguson convincingly demonstrates in relation to state bureaucracy in Lesotho, the capacity of the state to plan, even when it plans poorly, embeds a rationale and a cohort committed to the fate and reach of this state.^148^ While this can often be no more than the kernel of a path dependency, it undoubtedly places the capacity to frame and resolve social, economic and health concerns firmly with the state and its apparatus. The litter of forgotten plans which surface in the wake of international development denotes a set of collective endeavours. These at once produce the post-colonial state and reproduce its shortcomings; as Morten Jerven shows, poor statistics gain traction, narratives of deficiency and need are honed and relations of dependency – whether new or inherited from colonial times – take on their familiar shape.^149^

From our perspective, this effort on the part of planners and international organisations seems vain and ill-conceived. The economic difficulties that caught up with and swamped African states in the 1970s and 1980s had their roots in historical under-development, colonial mismanagement and a disordered transition to the exercise of self-government. The practice and fate of health planning in five nations in West and Central Africa in the 1960s exemplifies the processes, pressures and cast of characters implicated in the production of these difficulties. In its data poverty, rhetorical circularity and barely concealed political antagonisms across a variety of national, regional and global spheres, it captures key elements of a development paradigm which has come to be seen as flawed: technocratic, repressive, uninformative and unresponsive.

In spite of this, the paradigm persisted. Furnished by new metrics, more advanced and sensitive epidemiological and planning apparatus, expertise in operational research and a continually renovated set of targets and goals, planning in health for development has gathered strength over the past half century. The 1970s and 1980s saw a diminution in the capacity for autonomous development planning on the part of African states; all the same, along with the concomitant re-engineering of national health bureaucracies, the process carried on apace. Planning, as Ben-Amor and Clairmonte ruefully intone, is ‘a process that is historically irreversible’.^150^

## Footnotes

1.
World Health Organization, *Everybody’s Business: Strengthening Health Systems to Improve Health Outcomes: WHO’s Framework for Action* (Geneva: WHO, 2007).

2.
Josefien van Olmen, Bruno Marchal, Wim Van Damme, *et al.*, ‘Health Systems Frameworks in their Political Context: Framing Divergent Agendas’, *BMC Public Health*12, 1 (2012), 774; Marlee Tichenor and Devi Sridhar, ‘Universal Health Coverage, Health Systems Strengthening, and the World Bank’, *The BMJ*, 358, j3347 (2017).10.1186/1471-2458-12-774PMC354928622971107

3.

Frederick Cooper , Africa Since 1940: The Past of the Present (Cambridge: Cambridge University Press, 2002), 91–109.

4.
Randall Packard, *A History of Global Health: Interventions into the Lives of Other Peoples* (Baltimore: Johns Hopkins University Press, 2016), 133; Milton Roemer, ‘Priority for Primary Health Care: Its Development and Problems’, *Health Policy and Planning*, 1, 1 (1986), 58–66.10.1093/heapol/1.1.5810283014

5.
Nitsan Chorev, *The World Health Organisation between North and South* (Ithaca: Cornell University Press, 2012), 6–7; Milton Roemer, ‘Internationalism in medicine and public health’, in D. Porter (ed.), *The History of Public Health and the Modern State* (Amsterdam: Rodopi, 1994), 403–23, 417–21.

6.
John Farley, *Brock Chisholm, the World Health Organization, and the Cold War* (Vancouver: UBC Press, 2008), 111–18, 122–3; James Gillespie, ‘Social medicine, social security and international health’, in E. Rodríguez-Ocaña (ed.), *The Politics of the Healthy Life: An International Perspective* (Sheffield: EAHMH Publications, 2002), 219–39; K. Lee, *The World Health Organization (WHO)* (Oxford: Routledge, 2009), 14–17.

7.

John Thompson , ‘The geopolitical vision: the myth of an outmatched USA’, in J. Isaac and D. Bell (eds), Uncertain Empire. American History and the Idea of the Cold War (Oxford: Oxford University Press, 2012), 91–114.

8.
Randall Packard, ‘Malaria Dreams: Postwar Visions of Health and Development in the Third World’, *Medical Anthropology*, 17, (1997), 279–96; Socrates Litsios, ‘Malaria Control, the Cold War, and the Postwar Reorganization of International Assistance’, *Medical Anthropology*, 17, (1997), 255–78; Bob Reinhardt, *The End of a Global Pox: America and the Eradication of Smallpox in the Cold War Era* (Chapel Hill: UNC Press, 2015).

9.
WHO, *The First Ten Years of the World Health Organization* (WHO: Geneva, 1958), 334–51.

10.
Fraser Brockington, *World Health* (Edinburgh: Churchill Livingstone, 1975 [1958]), 175–85.

11.
WHO, *The Second Ten Years of the World Health Organization, 1958–67* (Geneva: WHO, 1968), 38, 40; see also M.Cueto, *The Value of Health: A History of the Pan American Health Organization* (Washington: PAHO, 2007), 131–2; J. Garcia Gutierrez, ‘Health Planning in Latin America’, *American Journal of Public Health*, 65, 10 (1975), 1047–9.10.2105/ajph.65.10.1047PMC17760131163699

12.
WHO, *ibid.*, 38–9; S. Litsios, *The Third Ten Years of the World Health Organization, 1968–77* (Geneva: WHO, 2008), 58–9.

13.
Litsios, *ibid,*, 11–13, 92–4, 117–31; S. Sapirie, ‘WHO and Health Planning: The Past, the Present and the Future’, *World Health Forum*, 19, (1998), 382–7, 382.10050164

14.
S. Hale Butterfield, *US Development Aid – An Historic First: Achievements and Failures in the Twentieth Century* (Westport: Praeger, 2004), 57–97; Jamey Essex, *Development, Security, and Aid: Geopolitics and Geoeconomics at the US Agency for International Development* (Athens: University of Georgia Press, 2013), 26–50.

15.
Herman Hilleboe, Arne Barkhuus and William Thomas, *Approaches to National Health Planning* (Geneva: WHO, 1972), 22, 29, 45, 69.

16.
Tony Judt, *Postwar: A History of Europe Since 1945* (London: Vintage, 2010 [2005]), 67; Glen O’Hara, *From Dreams to Disillusionment: Economic and Social Planning in 1960s Britain* (Basingstoke: Palgrave Macmillan, 2007).

17.
Richard Toye, *The Labour Party and the Planned Economy, 1931–51* (Woodbridge: Boydell Press, 2003), 4–5.

18.

Alec Nove , An Economic History of the USSR (Harmondsworth: Penguin, 1969), 136–159.

19.
Toye, *op. cit.* (note 17); Daniel Ritschel, *The Debate on Economic Planning in Britain in the 1930s* (Oxford: Clarendon Press, 1997); Political and Economic Planning, *Report on the British Health Services* (London: PEP, 1937), 16, 25, 230.

20.
David Ekbladh, ‘Meeting the Challenge from Totalitarianism: The Tennessee Valley Authority as a Global Model for Liberal Development, 1933–45’, *The International History Review*, 32, 1 (2010), 47–67; Michael J. Hogan, *The Marshall Plan: America, Britain and the Reconstruction of Western Europe, 1947–52* (Cambridge: Cambridge University Press, 1987).

21.
Joseph Hodge and Gerald Hödl, ‘Introduction’, in Joseph Hodge, Gerald Hödl and Martina Kopf (eds), *Developing Africa: Concepts and Practices in Twentieth-century Colonialism* (Manchester: Manchester University Press 2014), 1–34.

22.
Albert Sarraut, *La Mise en Valeur des Colonies Françaises* (Paris: Payot, 1923), 127; Frederick Lugard, *The Dual Mandate in British Tropical Africa* (Edinburgh: Blackwood, 1922) 175 and *passim*; H.W. Arndt, ‘Economic Development: A Semantic History’, *Economic Development and Cultural Change*, 29, 3 (1981), 457–66.

23.
Stephen Constantine, *The Making of British Colonial Development Policy 1914–40* (London: Frank Cass 1984) 157, 189.

24.
Michael Havinden and David Meredith, *Colonialism and Development: Britain and its Tropical Colonies, 1850–1960* (London: Routledge, 2002 [1993]), 195–205; Frederick Cooper, *Decolonization and African Society: The Labor Question in French and British Africa* (Cambridge: Cambridge University Press, 1996); Hodge and Hödl, *op. cit.* (note 21), 12–15; Christophe Bonneuil, ‘Development as Experiment: Science and State Building in Late Colonial and Postcolonial Africa, 1930–70’, *Osiris*, 15, (2000), 258–81.

25.
Author’s calculations: the 1929–40 sum is the average annual recommendation of Britain’s Colonial Development Advisory Committee; Constantine, *op. cit.* (note 23), 205; Havinden and Meredith, *ibid.*, 221–2; Jane K. Seymour, Martin Gorsky and Shakoor Hajat, ‘Health, Wealth and Party in Inter-War London’, *Urban History*, 44, 3 (2017), 464–91, at 475.

26.
Arturo Escobar, *Encountering Development: The Making and Unmaking of the Third World* (Princeton: Princeton University Press, 1995), 3–4; Wolfgang Sachs, ‘Introduction’, Gustavo Esteva, ‘Development’ and Arturo Escobar, ‘Planning’, in Wolfgang Sachs (ed.), *The Development Dictionary: A Guide to Knowledge as Power*, 2nd edn (London: Zed, 2010 [1992]), xv–xx, 1–23, 145–60; Gilbert Rist, *The History of Development: From Western Origins to Global Faith* (London: Zed, 2008 [1997]), 69–78; Harry S. Truman Library and Museum,‘Truman’s Inaugural Address, January 20, 1949’, URL:  https://www.trumanlibrary.org/whistlestop/50yr_archive/inagural20jan1949.htm (accessed 4 January 2018); Michael Hardt and Antonio Negri, *Empire* (London: Harvard University Press, 2000), 244–54.

27.
For summary, see A.G. Hopkins, ‘Development and the utopian ideal, 1960–99’, in Robin Winks and Alaine Low (eds), *The Oxford History of the British Empire: Volume V. Historiography* (Oxford: Oxford University Press, 1999), 635–52.

28.

W.W. Rostow , The Stages of Economic Growth (Cambridge: Cambridge University Press, 1960).

29.

Dean Tipps , ‘Modernization Theory and the Comparative Study of Societies: A Critical Perspective’, Comparative Studies in Society and History, 15, 2 (1973), 199–226.

30.

Sugata Bose , ‘Instruments and idioms of colonial and national development’, in Frederick Cooper and Randall Packard (eds), International Development and the Social Sciences: Essays on the History and Politics of Knowledge (Berkeley: University of California Press, 1997), 45–63.

31.
Havinden and Meredith, *op. cit.* (note 24), 252–8.

32.
Toyin Falola, *Development Planning and Decolonization in Nigeria* (Gainesville: University of Florida Press, 1996), xxii, 108, 127–39.

33.
Robert Tignor, *W. Arthur Lewis and the Birth of Development Economics* (Princeton: Princeton University Press, 2006); Edgar Dosman (ed.), *Raúl Prebisch: Power, Principle and the Ethics of Development* (Buenos Aires: Inter-American Development Bank, 2006).

34.

Eric Helleiner , ‘The Development Mandate of International Institutions: Where Did It Come From?’, Studies in Comparative International Development, 44, (2009), 189–211.

35.
David Webster, ‘Development Advisors in a Time of Cold War and Decolonization: The United Nations Technical Assistance Administration, 1950–59’, *Journal of Global History*, 6, 2 (2011), 249–72, 271; Amy Staples, *The Birth of Development* (Kent: Kent State University Press, 2006), 2–12.

36.
Benno Ndulu, ‘The evolution of global development paradigms and their influence on African economic growth’, in Benno Ndulu, Stephen O’Connell, Robert Bates, *et al.* (eds), *The Political Economy of Growth in Africa, 1960–2000* (Cambridge: Cambridge University Press, 2008), 315–47; William Easterly, *The White Man’s Burden: Why the West’s Efforts to Aid the Rest Have Done so Much Ill and So Little Good* (New York: Penguin, 2006); Paul Collier, *The Bottom Billion: Why the Poorest Countries are Failing and What Can Be Done About It* (Oxford: Oxford University Press, 2007).

37.
Andre Gunder Frank, *Capitalism and Underdevelopment in Latin America* (New York: Monthly Review Press, 1967); Immanuel Wallerstein, *The Capitalist World-Economy* (Cambridge: Cambridge University Press, 1979); Samir Amin, *Neo-Colonialism in West Africa* (New York: Monthly Review Press, 1973); Amin, *Maldevelopment: Anatomy of a Global Failure* (Cape Town: Pambazuka Press, 2011); Walter Rodney, *How Europe Underdeveloped Africa* (Washington: Howard University Press, 1982 [1972]).

38.
Escobar, *op. cit.* (note 26); James Ferguson, *The Anti-Politics Machine: Development, Depoliticization and Bureaucratic Power in Lesotho* (Cambridge: Cambridge University Press, 1990).

39.
Ferguson, *ibid.*, 3–23, 251–9; James C. Scott, *Seeing Like a State: How Certain Schemes to Improve the Human Condition Have Failed* (New Haven: Yale University Press, 1998).

40.
Claude Ake, *Democracy and Development in Africa* (Washington: Brookings Institution, 1996); Piotr Dutkiewicz and Gavin Williams, ‘All the King’s Horses and All the King’s Men Couldn’t put Humpty-Dumpty Together Again’, *IDS Bulletin*, 18, 3 (1987), 39–44.

41.

Morten Jerven , Poor Numbers: How We Are Misled by African Development Statistics and What to Do About It (Ithaca: Cornell University Press, 2013).

42.
For example, Dennis Ityavyar, ‘Background to the Development of Health Services in Nigeria’, *Social Science Medicine*, 24, 6 (1987), 487–99; Meredith Turshen, *The Political Ecology of Disease in Tanzania* (New Brunswick, NJ: Rutgers University Press, 1984).10.1016/0277-9536(87)90338-83296221

43.
Vicente Navarro, *Medicine Under Capitalism* (London: Croom Helm, 1976); Dennis Ityavyar, ‘Health Services Inequalities in Nigeria’, *Social Science Medicine*, 27, II (1988), 1223–35.10.1016/0277-9536(88)90352-83206253

44.
Sarah Hodges, ‘Second Opinion: The Global Menace’, *Social History of Medicine*, 25, 3 (2012), 719–28; Warwick Anderson, *Colonial Pathologies: American Tropical Medicine, Race, and Hygiene in the Philippines* (Durham: Duke University Press, 2006); Esteva, *op cit.* (note 26), 18; Escobar, *op. cit.* (note 26), 154.

45.
Ferguson, *op. cit.* (note 39), 209–12, is interested only in squabbles over resources, not the effectiveness of a planned health division in Lesotho.

46.
Angus Deaton, *The Great Escape: Health, Wealth and the Origins of Inequality* (Princeton: Princeton University Press, 2013); Samuel Preston, ‘The Changing Relation Between Mortality and Level of Economic Development’, *Population Studies*, 29, 2 (1975), 231–48.

47.
Scott, *op. cit.* (note 39), 325–7, 339–40.

48.
Stacy Leigh Pigg, “‘Found in most traditional societies”: traditional medical practitioners between culture and development’, in Cooper and Packard *op. cit.* (note 30), 259–90.

49.
US Department of State, ‘Address by President John F. Kennedy to the UN General Assembly’, September 25, 1961, at URL: http://www.state.gov/p/io/potusunga/207241.htm(accessed 4 January 2018).

50.
United Nations, *Five-Year Perspective, 1960–64: Consolidated Report on the Appraisals of the Scope, Trend and Costs of the Programmes of the United Nations, ILO, FAO, UNESCO, WHO, WMO and IAEA in the Economic, Social and Human Rights Fields* (Geneva: United Nations, 1960), 6, 20–1, 83–4.

51.

*Ibid.*, 60–62, 91. Gunnar Myrdal, *Economic Theory and Underdeveloped Regions* (London: Methuen, 1957).

52.
UN, *op. cit.* (note 50), 60.

53.

*Ibid.*, 62, 91.

54.
W.M. Louis, ‘Public enemy number one: the British Empire in the dock at the United Nations, 1957–71’, in Martin Lynn (ed.), *The British Empire in the 1950s: Retreat or Revival?* (Basingstoke: Palgrave Macmillan, 2006), 192–8.

55.
Chorev, *op. cit.* (note 5), 1–2.

56.

Iris Borowy , Coming to Terms with World Health. The League of Nations Health Organisation 1921–1946 (Frankfurt am Main: Peter Lang, 2009), 142.

57.
World Health Organization, *Expert Committee on Public Health Administration: First Report*, WHO Technical Report Series No. 55 (Geneva: WHO, 1952), 15.13006388

58.
Packard, *op. cit.* (note 8), 289; World Health Organization, *Methodology of Planning an Integrated Health Programme for Rural Areas: Second Report of the Expert Committee on Public Health Administration*, WHO Technical Report Series No. 83 (Geneva: WHO, 1954), 42–3.13169902

59.
Sapirie, *op. cit.* (note 13), 383.

60.
Sabine Clarke, ‘A Technocratic Imperial State? The Colonial Office and Scientific Research, 1940–60’, *Twentieth Century British History*, 18, 4 (2007), 460–4.

61.
See for example Albert Zahra, ‘Yaws Eradication Campaign in Nsukka Division, Eastern Nigeria’, *Bulletin of the World Health Organization*, 15, 6 (1956), 911–35.PMC253817413404467

62.
For further discussion, see Mary Dobson, Maureen Malowany and Robert Snow, ‘Malaria Control in East Africa: The Kampala Conference and the Pare-Taveta Scheme: A Meeting of Common and High Ground’, *Parassitologia*, 42, 1–2 (2000), 149–66.11234325

63.
World Health Assembly 14, WHA 14.58, *Declaration Concerning the Granting of Independence in Colonial Countries and Peoples and the Tasks of the World Health Organization* *, Thirteenth plenary meeting, 24 February 1961* (Geneva: WHO, 1961); World Health Assembly, 15, WHA15.57 *United Nations Development Decade* *, Thirteenth plenary meeting, 25 May 1962*, section 3 of the tenth report of the Committee on Programme and Budget (Geneva: WHO, 1962).

64.
WHO N5/372/3 Jacket 1 1962–73 USAID/WHO Co-ordination in the Field of National Health Planning, F. Grundy and M. Siegel, ‘Memorandum: Training of Consultants for National Health Planning in Africa’ 24 July 1962, 1; WHO N5/372/3 Jacket 1, ‘Minutes of the Regional Health Officers’ Meeting’, 2 August 1962, 1.

65.
WHO N5/372/3 Jacket 1, ‘Memorandum’, 24 July 1962, 2–3.

66.

*Ibid.*, 1.

67.
WHO N5/372/3 Jacket 1, ‘National Health Planning. Aide-memoire of meeting’, 24 April 1963; Memorandum, Israel to Schofield, ‘Brief on the Five African Plans’, 10 September 1973.

68.
WHO N5/372/3 Jacket 1, ‘National Health Planning. Aide-memoire’, 1–2.

69.

*Ibid.*, 3.

70.
World Health Organization Executive Board. EB 28/7, *Organizational Study on Co-ordination with the United Nations and the Specialized Agencies: Further Draft Submitted by the Director-General* (Geneva: World Health Organization, 1961), 21–2.

71.
WHO N5/372/3 Jacket 1, ‘Training of consultants for national health planning in Africa: Attachment’, 24 July 1962.

72.
WHO N5/372/3 Jacket 1, Sinai to Grundy, 3 August 1962; on Sinai: Odin Anderson, ‘The Politics of the Welfare State as Seen by Wilbur J. Cohen’, unpublished manuscript, 2012, 146; Anderson, ‘Influence of Social and Economic Research on Public Policy in the Health Field: A Review’, *Milbank Memorial Fund Quarterly*, 44, 3 (1966), 11–51, at 22–4.5338567

73.
WHO N5/372/3 Jacket 1, ‘Minutes of the Regional Health Officers’ Meeting’, 5.

74.

*Ibid.*, 3–4.

75.
WHO N5/372/3 Jacket 1, ‘National Health Planning. Aide-memoire’, 2.

76.

*Ibid*.

77.

*Op. cit.* (note 70), 7–8.

78.
International Labour Office, *The Cost of Medical Care: Studies and Reports 51* (Geneva: International Labour Office, 1959); B. Abel-Smith, *Paying for Health Services: A Study of the Costs and Sources of Finance in Six Countries* (Geneva: World Health Organization, 1963); Gillespie, *op. cit.* (note 6).

79.
WHO N5/372/3 Jacket 1, ‘National Health Planning. Aide-memoire’, 2.

80.
WHO N5/372/3 Jacket 1, ‘Progress Report on National Health Planning in Gabon, Liberia, Mali, Niger and Sierra Leone’, 1968, 1.

81.
WHO N5/372/3 Jacket 1, Baarkhuus to Curtis, 30 December 1964.

82.

*Op. cit.* (note 80), 2–4.

83.
See, for instance, Sierra Leone Ministry of Health, *National Health Plan, 1965–75* (Freetown: Ministry of Health, 1965), 26.

84.
WHO N5/372/3 Jacket 1, ‘National Health Planning. Aide-memoire’, 2.

85.
République du Niger, *Perspectives Décennales 1965–1974 de Développement des Services de Santé Publique de la République du Niger* (Niamey, 1964), 5. This language is used by Ibra Kabo, minister for public health, Niger.

86.

*Ibid.*, 22.

87.
WHO NHP/67.2, *Final Draft. Ten Year National Health Plan, 1966–67. Republic of Liberia*, 13–14.

88.
S. Jusu-Sheriff, minister of health, quoted in Sierra Leone, *op. cit.* (note 83), 7.

89.
République du Mali, *Plan Décennal (1er Juillet 1966–30 Juin 1976) De Développement des Services de Santé* (Bamako, 1966), and République du Niger, *op. cit.* (note 85).

90.

*Op. cit.* (note 83), Annex 1 and Addendum. These detail the establishment of a national laboratory service, and a restatement of health planning priorities, respectively.

91.

*Ibid*., 145.

92.
Jerven, *op. cit.* (note 41).

93.

*Op. cit.* (note 83), 17.

94.
WHO N5/372/3 Jacket 1, Memorandum, Israel to Schofield, ‘Brief of the Five African Plans’, 10 September 1973, Annex ‘Evaluation of the Five African Health Plans’, 2.

95.
The discussion of intertextuality draws on N. Fairclough, *Discourse and Social Change* (Cambridge: Polity, 1992), 101–36.

96.

*Op. cit.* (note 80), 4–5.

97.
WHO Technical Report Series No. 350, *National Health Planning in Developing Countries. Report of a WHO Expert Committee* (Geneva: WHO, 1967); WHO Technical Report Series No. 456, *Training in National Health Planning. Report of a WHO Expert Committee* (Geneva: WHO, 1970).4962815

98.
WHO N5/372/3 Jacket 1, Quenum to Karefa-Smart, ‘Memorandum: National Health Planning’, 25 April 1968; Quenum to Permanent Secretary, 31 July 1968.

99.
WHO N5/372/3 Jacket 1, Adjou-Moumouni to Chief NHP, ‘Memorandum National Health Plans for Five African Countries’, 31 July 1968.

100.
WHO N5/372/3 Jacket 1, Adjou-Moumouni to Chief NHP, 31 July 1968, ‘Sierra Leone Evaluation: National Health Plan’, 1.

101.
WHO N5/372/3 Jacket 1, Adjou-Moumouni to Chief NHP, 31 July 1968, ‘Evaluation (Mali) Plan National de Developpement’, 1.

102.
WHO N5/372/3 Jacket 1, Adjou-Moumouni to Chief NHP, 31 July 1968, ‘Liberia General Review of National Planning’.

103.
WHO N5/372/3 Jacket 1, Adjou-Moumouni to Chief NHP, 31 July 1968, ‘Evaluation Plan National de Developpement des Services de Sante Publique, Niger’, 1–2.

104.
WHO N5/372/3 Jacket 1, Karefa-Smart to Siegel, ‘Memorandum. Five National Health Plans in Africa: Gabon, Liberia, Mali, Niger and Sierra Leone’, 28 January 1969, 1.

105.
Amin, *Neo-Colonialism*, *op. cit.* (note 37), 227–36; Pascal James Imperator, *Mali: A Search for Direction* (London: Dartmouth, 1989), 58–64.

106.
James Barnes, *Gabon: Beyond the Colonial Legacy* (Boulder: Westview Press, 1992), 40–7, 71–3, 87–8.

107.
Ousmane Samab Mamadou and Mahaman Sani Yakabou, ‘Climate vulnerability, political instability, investment and growth in a landlocked, Sahelian economy’, in Benno Ndulu, Stephen O’Connell, Jean-Paul Azam, *et al.* (eds), *The Political Economy of Economic Growth in Africa, 1960–2000: Volume 2* (Cambridge: Cambridge University Press, 2008), 166–92.

108.

*Ibid.*, 176–7; Abdourahamne Idrissa and Samuel Decalo, *Historical Dictionary of Niger* (Lanham: Scarecrow Press, 2012), 8–9, 84–6; Virginia Thompson, ‘Niger’, in Gwendolen Carter (ed.), *National Unity and Regionalism in Eight African States* (Ithaca: Cornell University Press, 1966), 151–230.

109.
Cooper, *op. cit.* (note 3), 156–7; Robert Clower, George Dalton. Mitchell Harwitz, *et al.*, *Growth Without Development: An Economic Survey of Liberia* (Evanston: Northwestern University Press, 1966), 3–13.

110.
Elwood Dunn, Amos Beyan and Carl Patrick, *Historical Dictionary of Liberia* (Lanham: Scarecrow Press, 2001), xxviii–xxx.

111.
Colin Waugh, *Charles Taylor and Liberia* (London: Zed Books, 2011), 29–34, 56–7.

112.
Martin Kilson, *Political Change in a West African State: A Study of the Modernization Process in Sierra Leone* (Cambridge, MA: Harvard University Press, 1966), 203–16, 231–80; David Harris, *Sierra Leone: A Political History* (London: C. Hurst & Co., 2013), 45–6.

113.
Victor Davies, ‘Sierra Leone’s economic growth performance, 1961–2000’, in Ndulu, O’Connell, Azam *et al.* *op. cit.* (note 107), 660–95.

114.
Harris, *op. cit.* (note 112), 47–63.

115.
Thomas H. Lee, ‘Odyssey of a Homesick Healer’, *The Harvard Crimson*, 15 May 1974; John Karefa-Smart interview with Gary Schulze, Interview 1, Part 3, Interview 2, Part 2 https://www.youtube.com/watch?v=36AUcw3ASR0&nohtml5=False.

116.
Magbaily C. Fyle, *Historical Dictionary of Sierra Leone* (Lanham, Maryland: Scarecrow Press, 2006), 88–9.

117.
Fyle, *Historical Dictionary of Sierra Leone*, 88.

118.
Harris, *op. cit.* (note 112), 50–66; *Amnesty International Newsletter*, 1, 6 (1971), 2–3.

119.
John Karefa-Smart, ‘Africa and the United Nations’, *International Organization*, 19, 3 (1965), 764–73; quote: Lee, *op. cit.* (note 115).

120.
Mathurin C. Houngnikpo and Samuel Decalo, *Historical Dictionary of Benin* (Lanham: Scarecrow Press, 2013), 26–7; Adjou-Moumouni was also a national politician manqué, after his sweeping victory in Benin’s 1968 presidential election was forcibly annulled.

121.
WHO N5/372/3 Jacket 1, Cummings to Siegel, ‘Memorandum. Five National Health Plans in Africa: Gabon, Liberia, Mali, Niger and Sierra Leone’, 18 December 1968; Thomas Stuman, USAID Mission to Liberia, to Director-General, 3 November 1969.

122.
WHO N5/372/3 Jacket 1, Adjou-Moumouni to Chief NHP, ‘Memorandum National Health Plans for Five African Countries’, 14 October 1968.

123.
WHO N5/372/3 Jacket 1, Chief NHP to Director PHS, ‘Memorandum National Health Planning in African Region’, 19 February 1969, 1.

124.
WHO N5/372/3 Jacket 1, Karefa-Smart to Quenum, ‘National Health Plans’, 7 January 1969.

125.
WHO N5/372/3 Jacket 1, Siegel to Karefa-Smart, ‘Memorandum. Utilization of Balance of Funds Available under the Five National Health Plans in Africa: Gabon, Liberia, Mali, Niger and Sierra Leone’, 3 March 1969.

126.
WHO N5/372/3 Jacket 2 S. Chadha and J.E. Rizos, ‘Evaluation of the Planning Process in Liberia (National Health Planning)’, 16 April 1971, 10.

127.

*Ibid.*, 10, 18–19, 40.

128.

**Ibid.**, 27, 41.

129.

*Ibid.*, 41.

130.

*Ibid.*, 39.

131.
WHO N5/372/3 Jacket 1, Memorandum, Israel to Schofield, ‘Brief of the Five African Plans’, 10 September 1973, Annex ‘Evaluation of the Five African Health Plans’, 1.

132.
WHO N5/372/3 Jacket 1, ‘Evaluation of the Five African Health Plans’, 1.

133.
Hilleboe *et al.* *op. cit.* (note 15), 13–14.

134.
WHO N5/372/3 Jacket 1, ‘Evaluation of the Five African Health Plans’, 2.

135.
WHO N5/372/3 Jacket 1, Kemp to Schofield, 2; ‘Memorandum’, Litsios to Director SHS, 1 October 1973.

136.
Akin Mabogunje, ‘Urban Planning and the Post-Colonial State in Africa: A Research Overview’, *African Studies Review*, 33, 2 (1990), 124.

137.
Chorev, *op. cit.* (note 5), 13.

138.
WHO N5/372/3 Jacket 1, ‘Meeting of the Regional Health Officers’ Meeting’, 1.

139.

Abdelmalek Bel-Amor and Frederick Clairmonte , ‘Planning in Africa’, Journal of Modern African Studies, 3, 4 (1965), 475.

140.
Hilleboe *et al.*, *op. cit.* (note 15), 13–14.

141.

*Op. cit.* (note 139), 494.

142.

*Ibid.*, 477.

143.

T.A. Lambo and S.B. Day , Issues in Contemporary International Health (New York: Springer, 1980), 1–9.

144.
James Webb and Tamara Giles-Vernick, ‘Introduction’, in Giles-Vernick and Webb (eds), *Global Health in Africa: Historical Perspectives on Disease Control* (Athens, OH: Ohio University Press, 2013), 3–7.

145.
William Easterly, ‘Introduction: can’t take it anymore?’, in William Easterly (ed.), *Reinventing Foreign Aid* (Cambridge, MA: MIT Press, 2008), 1–43, at 6.

146.
WHO N5/372/3 Jacket 1, ‘Evaluation of the Five African Health Plans’, 2.

147.
James Ferguson, *Global Shadows: Africa in the Neoliberal World Order* (Durham, NC: Duke University Press, 2006), 19.

148.
Ferguson, *op. cit.* (note 38).

149.

*Op. cit.* (note 41).

150.

*Op. cit.* (note 139), 493.

